# Association between MIC-1 and Type 2 Diabetes: A Combined Analysis

**DOI:** 10.1155/2019/7284691

**Published:** 2019-11-16

**Authors:** Jianan Lu, Yue Zhang, Xingxuan Dong, Jiawen Lu, Chen Zhang, Jieyu Liu, Qingzhou Yu, Haoyue Teng, Qingkui Yao, Jieyun Yin, Liqiang Qin

**Affiliations:** ^1^Department of Epidemiology and Biostatics, Jiangsu Key Laboratory of Preventive and Translational Medicine for Geriatric Diseases, School of Public Health, Medical College of Soochow University, 199 Ren Ai Road, Suzhou, China; ^2^Community Health Service Center of Minglou, Subdistrict Jiangdong District, Ningbo, China; ^3^Department of Nutrition and Food Hygiene, School of Public Health, Soochow University, Suzhou, China

## Abstract

**Background and Objectives:**

Type 2 diabetes mellitus (T2DM) is an epidemic disease that endangers human health seriously. Recently, a large number of reports have revealed that macrophage-inhibiting cytokine-1 (MIC-1) is linked with T2DM, but the results were inconclusive. The aim of this study was to perform bioinformatics analysis of the association between MIC-1 and T2DM.

**Material and Methods:**

Datasets and relevant literatures were searched in Gene Expression Omnibus (GEO), PubMed, Google Scholar, and Web of Science till September 20, 2019. Expression levels of MIC-1 were extracted, pooled, and compared between T2DM cases and controls.

**Results:**

In summary, 11 GEO datasets and 3 articles with 421 T2DM cases and 711 controls were finally included. The expression level of MIC-1 was significantly higher in T2DM patients compared with controls, with a standard mean difference (SMD) of 0.54 and a 95% confidence interval (95% CI) of 0.24-0.83; in blood samples, the difference was still significant (SMD = 0.65; 95%CI = 0.24‐1.06). Meanwhile, the expression level of *MIC-1* plays a significant role in differentiating T2DM cases from controls; the combined sensitivity, specificity, and odds ratio were 0.83 (95%CI = 0.72‐0.90), 0.59 (95%CI = 0.45‐0.72), and 1.64 (95%CI = 1.35‐1.99), respectively. The summary receiver operating characteristic (SROC) curve demonstrated that the area under the curve (AUC) was 0.81 (95%CI = 0.77‐0.84).

**Conclusion:**

Our results suggested that the expression levels of MIC-1 were significantly higher in T2DM patients in multiple tissues including blood samples.

## 1. Introduction

Diabetes is a global disease which is described as a type of metabolic disorder distinguished by increased blood glucose concentration. At present, nearly half a billion people suffer from diabetes [1], and the number of people affected with diabetes has been rising for the last decades [2]. Type 2 diabetes mellitus (T2DM) is the most common type of diabetes and accounts for around 90% of total diabetes cases [3]. Overweight and obesity have been regarded as the main risk factors contributing to T2DM [4]. Approximately 50% of obese subjects will develop into T2DM [5]. Thus, pathway targeting energy metabolism may provide useful diagnosis and treatment information for T2DM [6].

Macrophage-inhibiting cytokine-1, also termed growth differentiation factor 15 (GDF-15), encodes a secreted protein of transforming growth factor beta (TGF-beta) family [7]. It is weakly expressed in multiple tissues under normal conditions [7]. The upregulated production of MIC-1 is triggered in response to inflammation such as tissue injury, biomechanical stress, and anoxia [8, 9]. MIC-1 has been considered to play a pivotal role in the development and progression of many diseases such as cardiovascular diseases and malignant cancer [10–12]. Recently, MIC-1 is considered as a long-term metabolism regulator with a function of increasing lipolysis as an adipokine in a paracrine fashion [13–15]. There have been evident findings in mice that overexpression of MIC-1 could lower the preference for fat intake and improve glucose tolerance and insulin sensitivity compared with MIC-1-null mice which further manifests its protective role in energy homeostasis [16–18].

Although several studies have depicted the specific role of MIC-1 in energy expenditure and metabolic activities [13, 14, 19–22], current findings clarifying the relationship between MIC-1 and T2DM are still rare. In our study, we conducted a meta-analysis based on a combination of GEO datasets and relevant clinical reports to illustrate the association between MIC-1 expression levels and T2DM.

## 2. Materials and Methods

### 2.1. Data Acquisition and Search Strategy

A well-established database of microarray was searched for the current meta-analysis up to September 20, 2019: the Gene Expression Omnibus (GEO; https://www.ncbi.nlm.nih.gov/geo/). The search strategy was as follows: (“diabetes mellitus” OR “diabetes insipidus” OR DIABETES) AND “Homo sapiens”.

Afterwards, a systematic literature was searched in PubMed, Google Scholar, and Web of Science, using the combination of key words “diabetes” AND “MIC-1”. The language of relevant articles was restricted to English. In order to find more qualified literatures, we viewed the reference lists of publications selected for inclusion.

**Figure 1:**
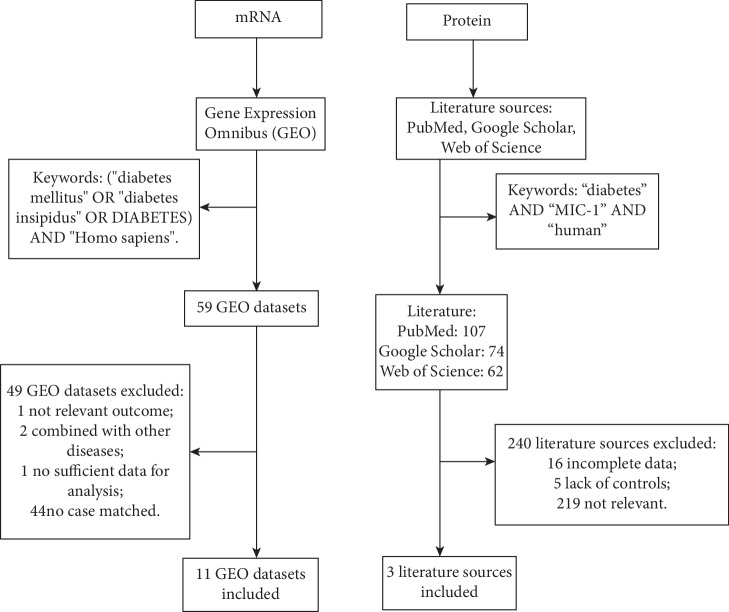
Search flow diagram for literature selection.

The process on how we conducted our data search and acquisition of the statistics was described in Figure 1.

### 2.2. Inclusion and Exclusion Criteria

The inclusion criteria of datasets and suitable literature were as follows: (1) the expression levels of MIC-1 were compared between T2DM patients and nondiabetes people; (2) each study should have at least 10 samples; (3) the original expression profiling data of MIC-1 and its mean and standard deviation (SD) should be offered or could be calculated; (4) samples should be collected from humans.

Studies were excluded if (1) studies have examined other types of diabetes such as type 1 diabetes; (2) studies or data are about animals or cell line; and (3) samples have overlapped with other studies.

### 2.3. Quality Control and Data Extraction

Two authors (Yue Zhang and Jianan Lu) extracted the information from all qualified datasets in line with the inclusion criteria independently, and any problem or ambiguity was discussed with the team. The following information was extracted for every dataset: last name of first author, country of origin, published year, study subjects (disease status and sample type), expression values, means, and SD of MIC-1.

### 2.4. Statistical Analysis

At first, we extracted MIC-1 expression levels from datasets and qualified literature; mean and SD were calculated. Next, we performed a meta-analysis to describe expression difference. Forest plots were used to get pooled standard mean difference (SMD) and 95% confidence interval (95% CI). We tested the possibility of heterogeneity by Cochran's *Q*-statistic and *I*^2^ statistics. A *P* value < 0.05 and an *I*^2^ > 50% were considered as heterogeneous, and the random effect model would be chosen to calculate the pooled SMD [23]. Otherwise, the fixed effect model would be selected. Besides, funnel plots and Begg's test were used to check the potential publication bias. Obesity is usually classified by body mass index: BMI ≥ 30 as obesity and BMI < 30 as nonobesity [24]. As the original data from the literatures were not available, we only included *MIC-1* expression data from GEO datasets and then conducted diagnostic analysis to assess the diagnostic possibility of *MIC-1* in T2DM patients. We carried out a meta-analysis with SROC to check the expression level of *MIC-1* in T2DM patients. Additionally, multivariate analysis of covariance (MANCOVA) was used to test differences in *MIC-1* between T2DM and non-T2DM groups with age and BMI as covariates in qualified dataset. A nominal level of significance *P* < 0.05 was accepted.

The data analyses were performed by SAS (SAS Institute Inc., NC, USA) and STATA (STATA Corporation, College Station, TX, USA).

## 3. Results

### 3.1. Characteristics of Included Studies

After initial searching from titles and content of abstract, 11 related datasets (GSE9006, GSE20966, GSE12643, GSE13760, GSE16415, GSE23343, GSE25724, GSE38642, GSE26168, GSE19420, and GSE27951) as well as 3 articles were obtained. Finally, a total of 14 studies including 421 T2DM patients and 711 nondiabetes samples were included for the current meta-analysis (Table 1).

### 3.2. Meta-Analysis of MIC-1 Expression in T2DM Patients and Controls

**Figure 2:**
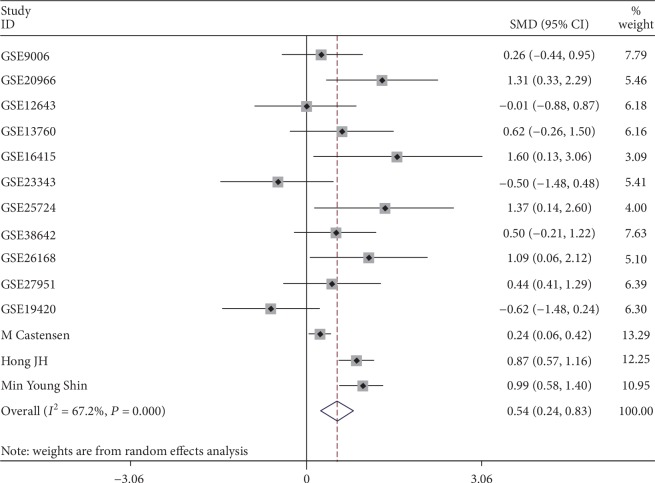
Forest plot showing SMD of MIC-1 expression between T2DM patients and nondiabetes people. The random effect model was used in all groups.

**Figure 3:**
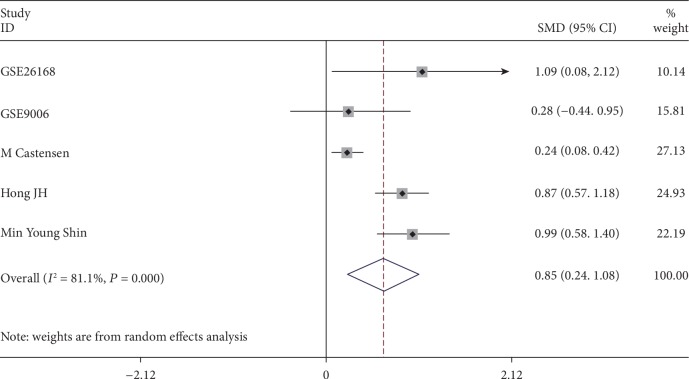
Forest plot showing SMD of MIC-1 expression in blood samples between T2DM patients and nondiabetes people. The random effect model was used in all groups.

**Figure 4:**
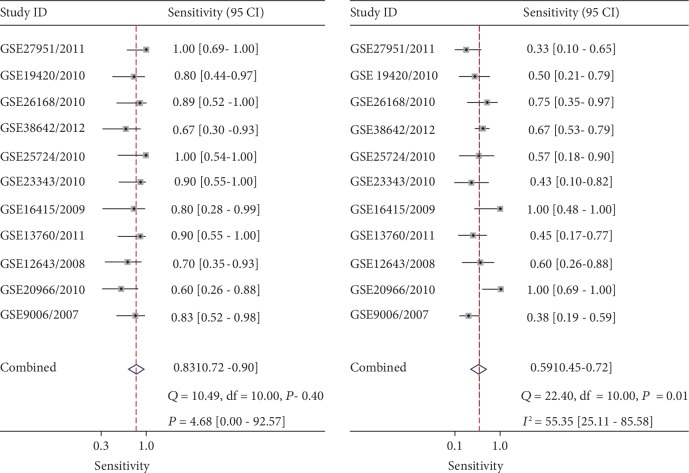
Diagnostic analysis of *MIC-1* value in T2DM patients.

**Figure 5:**
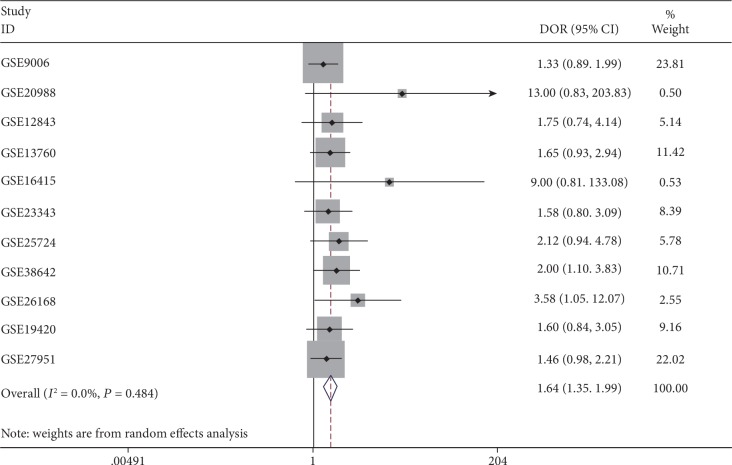
Summary receiver operating characteristic curve of *MIC-1* value in T2DM patients.

**Figure 6:**
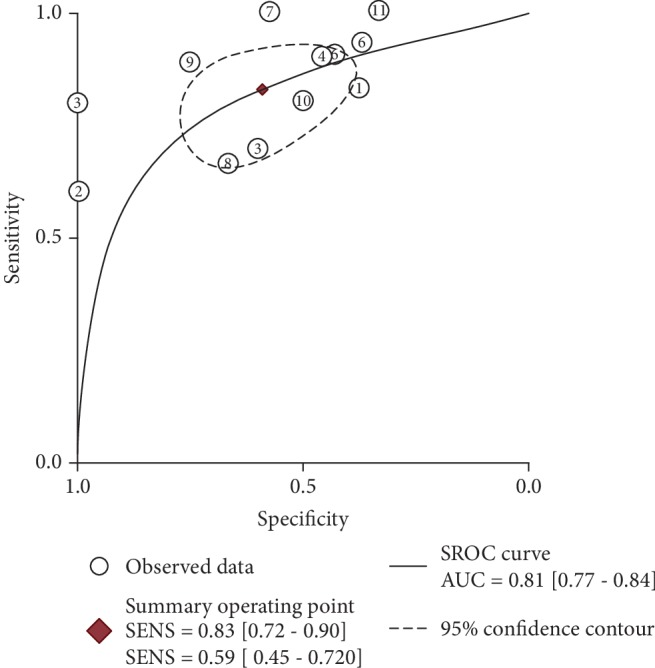
Sensitivity analysis of the value of *MIC-1* in T2DM patients.

It was found that the expression of MIC-1 was significantly increased in T2DM patients compared with controls, with SMD of 0.54 (95%CI = 0.24‐0.83) (Figure 2). Additionally, expression levels of MIC-1 in the blood were obviously higher with SMD of 0.65 (95%CI = 0.24‐1.06) (Figure 3). There is no publication bias (*P* = 0.511), and the result remained stable according to the sensitivity analysis (Supplementary [Supplementary-material supplementary-material-1] and Supplementary [Supplementary-material supplementary-material-1]). Subsequently, a diagnostic test was performed to evaluate the diagnostic effect of *MIC-1* for T2DM patients. Figures 4 and 5 showed the combined sensitivity, specificity, and odds ratio, the corresponding values were 0.83 (95%CI = 0.72‐0.90), 0.59 (95%CI = 0.45‐0.72), and 1.64 (95%CI = 1.35‐1.99), respectively. The SROC curve (Figure 6) demonstrated that the area under the curve (AUC) was 0.81 (95%CI = 0.77‐0.84).

### 3.3. MIC-1 and BMI in T2DM

It is of great value to study cardiovascular risk factors in T2DM. Among the included 11 GEO datasets, there were 5 datasets (GSE38642, GSE25724, GSE19420, GSE20966, and GSE27951) with 45 T2DM patients and 95 controls reported information about age and BMI. By utilizing MANCOVA and controlling for age and BMI, the corresponding least-square means of MIC-1 in T2DM patients and controls were shown in Supplementary [Supplementary-material supplementary-material-1]. However, the combined difference of least-square means of *MIC-1* between T2DM and normal controls was not significant in the random model with a SMD of 0.78 (95%CI = −0.97‐2.53) (Supplementary [Supplementary-material supplementary-material-1]).

We also stratified T2DM patients into two groups based on BMI (BMI ≥ 30: obese; BMI < 30: nonobese) in 4 qualified datasets (GSE38642, GSE19420, GSE20966, and GSE27951), which include 25 obese and 21 nonobese T2DM patients (Supplementary [Supplementary-material supplementary-material-1]). Compared with T2DM patient that are normal or overweight, obese T2DM patients tended to have lower levels of *MIC-1*, with a SMD of -0.42 (95%CI = −1.09‐0.25), although it failed to reach significance (Supplementary [Supplementary-material supplementary-material-1]).

## 4. Discussion

In our report, MIC-1 value increased in different tissues of T2DM patients compared with nondiabetes people, including blood samples.

MIC-1 has been suggested to be linked with obesity and T2DM. Accumulating evidence in animals clarifies that the overexpression of MIC-1 leads to weight loss, enhanced insulin sensitivity, and higher glucose tolerance. It is assumed that MIC-1 might keep a protective factor in obese and obesity-related diseases like T2DM [25]. Firstly, previous publication has elucidated that MIC-1 acts on feeding centers in the hypothalamus and brainstem to regulate weight control [26]. Secondly, MIC-1 is a secretory product of adipocytes and plays a role in improving lipolysis which is reversely correlated with serum total cholesterol [20, 27]. Moreover, elevated MIC-1 expression could invoke improved insulin activity and mediate Th2 cytokines like IL-13 function [14, 16]. Previous studies in human etiology of T2DM have demonstrated that high glucose would promote cellular aging and apoptosis. Acting as a protective regulator, MIC-1 expression is accordingly elevated in endothelial cells by ROS- and p53-dependent pathway [28]. MIC-1 is acknowledged as an anti-inflammation cytokine and responds positively to chronic inflammatory disease like T2DM. This acknowledgment may be the reason of higher MIC-1 level in T2DM. Also, a hypothesis was proposed that MIC-1 may function as a self-guarded cytokine in T2DM accounting for reduced food intake and then weight loss. After the peak of MIC-1 expression, it is not sufficient to compensate in the long-term low-grade inflammation, leading to an unstoppable progression of weight gain and metabolism dysfunction [29]. This hypothesis is consistent with our finding that MIC-1 was found to have lower expression levels in obese patients compared with nonobese ones.

Importantly, evidence showed that the serum MIC-1 level ranks the highest in T2DM patients, intermediate in individuals with prediabetes, and lowest in patients without diabetes [20, 30–32]. Elevated serum MIC-1 expression also suggested a high risk for T2DM-related complications including cardiovascular risks [33, 34] and renal diseases [35, 36]. The current study found a positive correlation between T2DM and MIC-1 level in blood samples, suggesting that MIC-1 could be a practical research target in the future.

Interestingly, an experiment in mice has reported that treating wild-type mice with both MIC-1 and liraglutide, a long-term glucagon-like peptide 1 (GLP-1) agonist, shows a synergistic impact on weight loss [18]. Progress has been made in combined utility of MIC-1 treatment and mature clinical medicine which shows a promising route for MIC-1 as a therapy biomarker. A fair number of progress have been made in identifying GFRAL, an orphan receptor of glial-derived neurotropic factor receptor *α*, as a receptor for MIC-1 and its neuronal circuits in which it acts [22, 37–39]. Based on this breakthrough, a class of MIC-1/GFRAL/RET-based drugs can be highly anticipated for treatment in T2DM [40].

The limitation of our study should also be of concern. First, our study estimated the contribution of numbered risk factors accounting for T2DM, such as age and BMI. Due to the limited data we obtained, we were unable to remove other influential factors such as smoking, dietary habit, and sedentary lifestyle. Second, our conclusion is summarized by a meta-analysis, causal relationship cannot be confirmed. Therefore, laboratory experiments should be performed to address the comprehensive mechanism between MIC-1 and T2DM.

## 5. Conclusion

Our study suggested that the expression level of MIC-1 was significantly higher in T2DM patients in multiple tissues including blood samples.

## Figures and Tables

**Table 1:** Characteristics of MIC-1 expression profiling datasets included in the current meta-analysis between T2DM and non-T2DM population.

Dataset	Country and publication year	Sample type	Platform	Tested substance	Case		Control	
					Sample size	MIC-1 mean and SD	Sample size	MIC-1 mean and SD
GSE20966	USA, 2010	Pancreatic beta cell	GPL1352	mRNA	10	5.647 ± 3.367	10	2.172 ± 1.655
GSE12643	Denmark, 2008	Myotubes	GPL8300	mRNA	10	10.345 ± 0.229	10	10.348 ± 0.408
GSE13760	Denmark, 2011	Arterial tissue (intima media)	GPL571	mRNA	10	101.222 ± 9.536	11	95.791 ± 7.878
GSE16415	India, 2009	Omentum (visceral tissue) tissue	GPL2986	mRNA	5	0.736 ± 0.521	5	0.136 ± 0.098
GSE23343	Japan, 2010	Liver	GPL570	mRNA	10	0.350 ± 0.118	7	0.438 ± 0.240
GSE25724	Italy, 2010	Pancreatic islets	GPL96	mRNA	6	4.660 ± 0.182	7	4.363 ± 0.242
GSE38642	Sweden, 2012	Pancreatic islets	GPL6244	mRNA	9	10.000 ± 0.512	54	9.691 ± 0.626
GSE19420	Netherlands, 2010	Skeletal muscle biopsies	GPL570	mRNA	10	3.424 ± 0.194	12	3.632 ± 0.416
GSE26168	Singapore, 2010	Blood	GPL6883	mRNA	9	8.278 ± 7.719	8	−3.150 ± 12.954
GSE9006	USA, 2007	Blood	GPL96	mRNA	12	19.225 ± 17.956	24	15.308 ± 13.606
GSE27951	UK, 2011	Adipose tissue	GPL570	mRNA	10	4.542 ± 0.271	12	4.403 ± 0.343
Castensen [41]	Germany, 2010	Blood		Protein	180	537.100 ± 166.440	372	499.700 ± 149.330
Hong et al. [30]	Korea, 2014	Blood		Protein	75	866.040 ± 628.210	137	484.050 ± 291.000
Shin et al. [34]	Korea, 2016	Blood		Protein	65	643.290 ± 535.390	42	210.120 ± 211.810

## Abbreviations

T2DM: Type 2 diabetes mellitus
MIC-1: Macrophage-inhibiting cytokine-1
GEO: Gene Expression Omnibus
SMD: Standard mean difference
SROC: Summary receiver operating characteristic
GFRAL: Glial-derived neurotropic factor receptor *α*
BMI: Body mass index
AUC: Area under the curve
MANCOVA: Multivariate analysis of covariance.

## Supplementary Materials

Supplementary Materials Supplementary Figure S.1: Begg's funnel plot for the assessment of potential publication bias in the samples. *P* for Begg's test is 0.511 (continuity corrected). Supplementary Figure S.2: sensitivity analysis of the value of MIC-1 in T2DM patients. Supplementary Figure S.3: forest plot showing the differences in least-square means between T2DM and non-T2DM. Random model was used to test the differences. Supplementary Figure S.4: forest plot showing the SMD of MIC-1 expression between obese and nonobese T2DM patients. Fixed effect model was used in all groups. Supplementary Table S.1: characteristics of age, BMI, and MIC-1 expression levels by two groups (T2DM and non-T2DM). Supplementary Table S.2: characteristics of MIC-1 expression profiling datasets between obese and nonobese T2DM patients.
