# Supplementary material for: Association between MIC-1 and Type 2 Diabetes: A Combined Analysis
Source: Dis Markers. 2019 Nov 16;2019:7284691. doi: 10.1155/2019/7284691 (PMC6885201; doi:10.1155/2019/7284691)
Supplement: Supplementary Materials — Supplementary Figure S.1: Begg's funnel plot for the assessment of potential publication bias in the samples. P for Begg's test is 0.511 (continuity corrected). Supplementary Figure S.2: sensitivity analysis of the value of MIC-1 in T2DM patients. Supplementary Figure S.3: forest plot showing the differences in least-square means between T2DM and non-T2DM. Random model was used to test the differences. Supplementary Figure S.4: forest plot showing the SMD of MIC-1 expression between obese and nonobese T2DM patients. Fixed effect model was used in all groups. Supplementary Table S.1: characteristics of age, BMI, and MIC-1 expression levels by two groups (T2DM and non-T2DM). Supplementary Table S.2: characteristics of MIC-1 expression profiling datasets between obese and nonobese T2DM patients. [file 7284691.f1.docx]

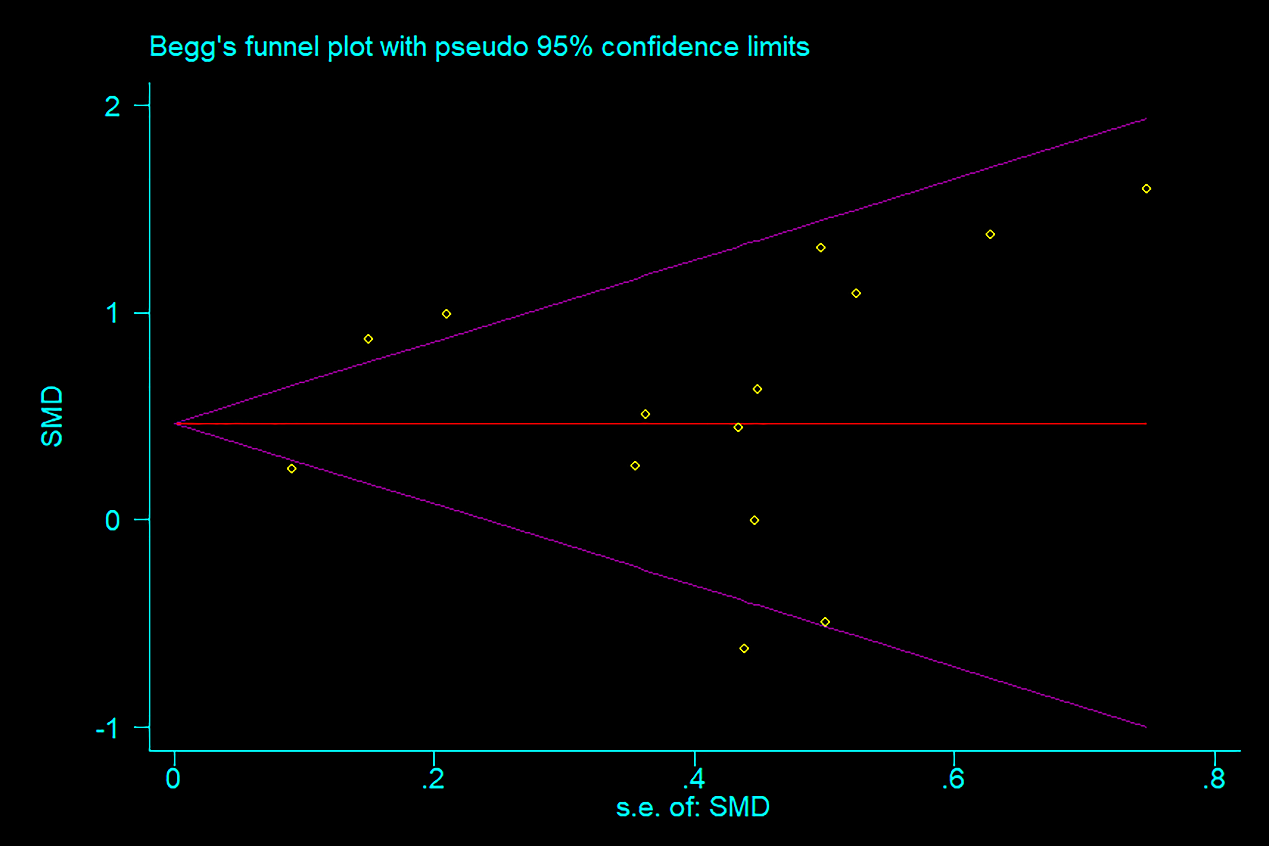


Figure S.1: Begg’s funnel plot for the assessment of potential publication bias in the samples. P for Begg’s test is 0.511 (continuity corrected).


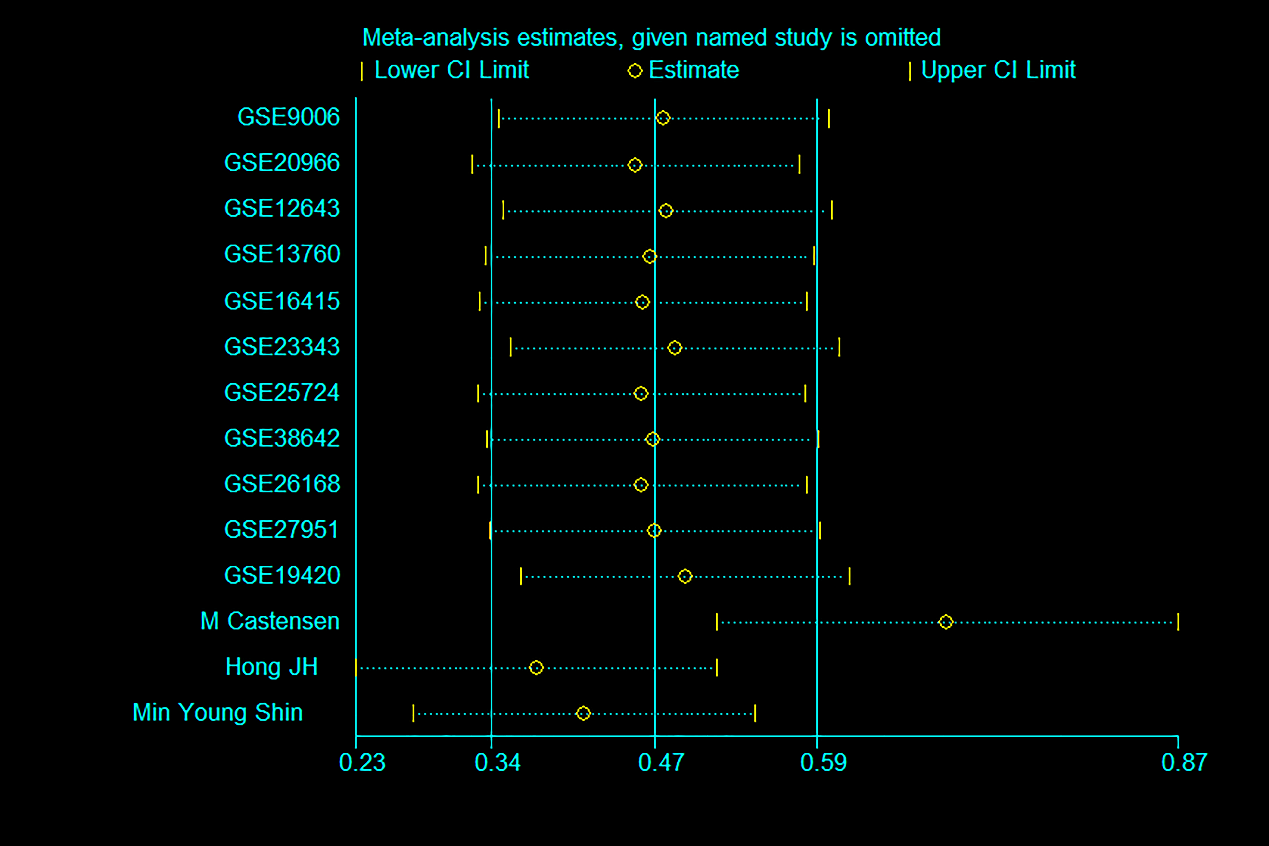


Figure S.2: Sensitivity analysis of the value of MIC-1 in T2DM patients.


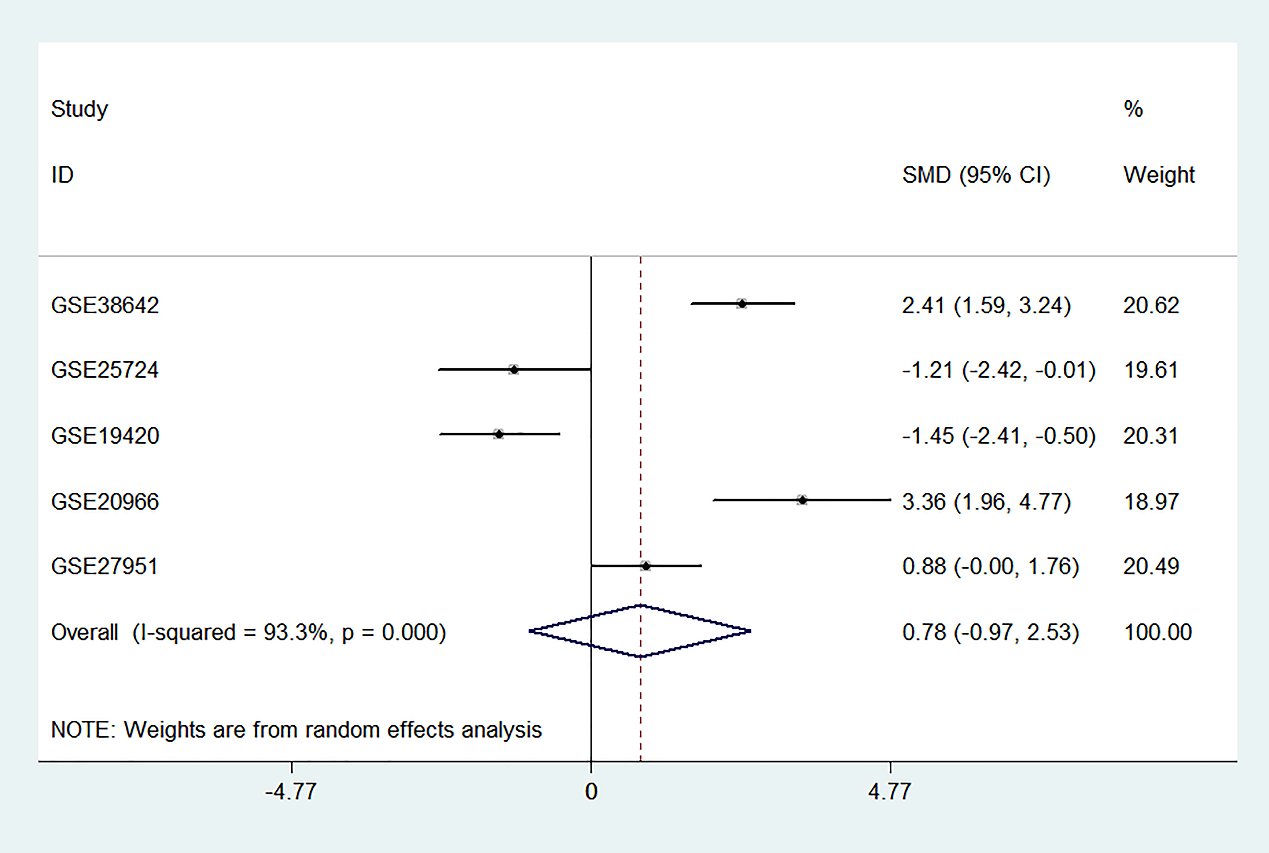


Figure S.3: Forest plot showing the differences in least-square means between T2DM and non-T2DM. Random model was used to test the differences.


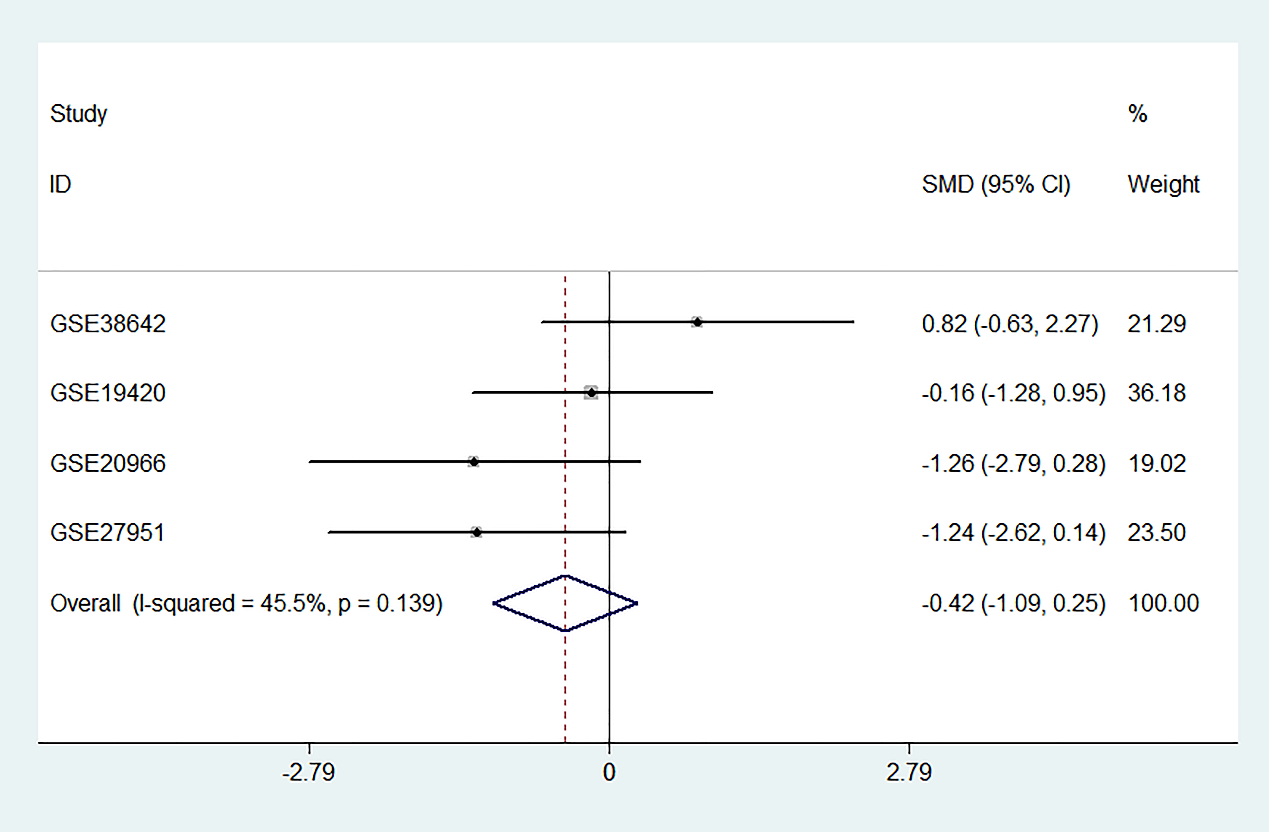


Figure S.4: Forest plot showing the SMD of *MIC-1* expression between obese and non-obese T2DM patients. Fixed effect model was used in all groups.

Table S.1**:** Characteristics of age, BMI, *MIC-1* expression levels by two groups (T2DM and Non-T2DM).

| GEO Datasets | Case/Control | Age Mean (SD) | |  | BMI Mean (SD) | |  | MIC-1 value Mean (SD) | |  | Least-square Means (SD) | | *P* value |
| --- | --- | --- | --- | --- | --- | --- | --- | --- | --- | --- | --- | --- | --- |
|  |  | Case | Control |  | Case | Control |  | Case | Control |  | Case | Control |  |
| GSE38642 | 9/54 | 57.000(13.115) | 56.611(9.758) |  | 28.500(4.695) | 25.954(3.485) |  | 10.000(0.512) | 9.691(0.626) |  | 9.964(0.212) | 9.698(0.085) | 0.250 |
| GSE25724 | 6/7 | 70.500(9.182) | 58.143(17.286) |  | 26.000(2.205) | 24.829(2.514) |  | 4.660(0.182) | 4.363(0.242) |  | 7.952(0.762) | 8.837(0.700) | 0.438 |
| GSE19420 | 10/12 | 60.000(6.903) | 56.083(5.992) |  | 32.272(3.484) | 32.943(4.610) |  | 3.424(0.194) | 3.632(0.416) |  | 3.450(0.116) | 3.610(0.105) | 0.332 |
| GSE20966 | 10/10 | 67.667(7.246) | 60.636(4.739) |  | 30.856(6.161) | 30.630(5.189) |  | 5.647(3.367) | 2.172(1.655) |  | 5.405(0.890) | 2.414(0.890) | 0.035 |
| GSE27951 | 10/12 | 54.800(6.268) | 45.583(13.222) |  | 32.760(8.537) | 30.100(6.016) |  | 4.542(0.271) | 4.403(0.343) |  | 4.514(0.104) | 4.427(0.095) | 0.552 |

Table S.2: Characteristics of *MIC-1* expression profiling datasets between obese and non-obese T2DM patients.

| Dataset | Country and Public Year | Sample Type | Platform | Tested  Substance | Obese | |  | Non-obese | |
| --- | --- | --- | --- | --- | --- | --- | --- | --- | --- |
|  |  |  |  |  | Sample Size | MIC-1 mean and SD |  | Sample Size | MIC-1 mean and SD |
| GSE38642 | Sweden,2012 | Pancreatic islets | GPL6244 | mRNA | 3 | 10.290±0.239 |  | 6 | 9.855±0.611 |
| GSE27951 | UK,2011 | Adipose tissue | GPL570 | mRNA | 5 | 4.388±0.179 |  | 5 | 4.696±0.302 |
| GSE20966 | USA,2010 | Pancreatic beta cell | GPL1352 | mRNA | 3 | 3.780±4.367 |  | 6 | 7.317±1.856 |
| GSE19420 | Netherlands,2010 | Skeletal muscle biopsies | GPL570 | mRNA | 14 | 3.395±0.176 |  | 4 | 3.425±0.206 |
